# Supplementary material for: Self-Reported Dietary Choices and Oral Health Care Needs during COVID-19 Quarantine: A Cross-Sectional Study
Source: Nutrients. 2022 Jan 13;14(2):313. doi: 10.3390/nu14020313 (PMC8779450; doi:10.3390/nu14020313)
Supplement: Supplementary file 1 [file nutrients-14-00313-s001.zip › nutrients-1545444-supplementary.pdf]

The study is carried out solely for scientific purposes by: Poznan University of Medical Sciences, Uniwersytet Medyczny im. Karola Marcinkowskiego w Poznaniu

PLEASE GIVE BACK THIS QUESTIONNAIRE TO VOLONTEER!!!! THANK YOU!!!!

Table S1: Self-designed questionnaire.

1	**Did you suffer from COVID-19 in 2020/2021?**	□ YES □ NO
2	**Have you been sent to quarantine in 2020/2021?**	□ YES □ NO
3	***Did you work remotely due to pandemic reasons in 2020/2021?***	□ YES □ NO
4	**Did you have any dental appointment in 2020/2021?**	□ YES □ NO
5	**How many dental visits did you have in 2020/2021?**	0 □ teledentistry 1 2 3 4 □ more
6	**Were you afraid of a dental visit from epidemic reasons in 2020/2021?**	□ YES □ NO
7	**What were the reasons for a dental visit in 2020/2021?**<br>(you can choose more than 1 answer if needed)	□ NO<br>□ toothache<br>□ gingivitis (pain/bleeding/swelling of gums)<br>□ dental examination (routine and non-invasive)<br>□ continuation of dental treatment<br>□ conservative treatment<br>□ surgical treatment<br>□ prosthetic treatment<br>□ orthodontic treatment<br>□ oral/tooth trauma<br>□ oral mucosa treatment
8	Was there any problem with arranging a dental appointment due to the pandemic?	□ YES □ NO
9	Did you postpone your dental appointment because of the pandemic?	□ YES □ NO
10	**Do you currently have complaints in mouth?**<br>(whether you have any complaints in oral cavity?	□ toothache □YES □ NO<br>□ gums bleeding □YES □ NO<br>□ stains/calculus □YES □ NO<br>□ lost of filling □YES □ NO<br>□ tooth hypersensitivity □YES □ NO
11	**Are you currently concerned about a dental visit due to the pandemic?**	□ YES □ NO
12	**Has the frequency of your meals increased daily?**	□ NO x2 x3 x4 >5
13	**Has the amount of consumed sweets/ sweet snacks increased per day?**	□ NO x2 x3 x4 >5
14	**Has the frequency of drinking a certain drink increased in 2020/2021? Whose?**	□ YES □ whose?.....……… □ NO
15	**Do you sweeten hot drinks with sugar?**	□ YES □ NO
16	**What is the time of the last meal in the evening**	18:00 20:00 22:00 24:00

The study is carried out solely for scientific purposes by: Poznan University of Medical Sciences, Uniwersytet Medyczny im. Karola Marcinkowskiego w Poznaniu

PLEASE GIVE BACK THIS QUESTIONNAIRE TO VOLONTEER!!!! THANK YOU!!!!

	**before bedtime?**	
17	**Do you smoke cigarettes? How many times a day?**	□ NO □ YES, SMOKE < packet a day<br>□ YES, SMOKE > packet a day
18	**How often do you drink alcohol?**	□ each day<br>□ several times a week<br>□ 1x week<br>□ occasionally (only special occasions, e.g. birthdays, holidays)<br>□ I don't drink at all
19	**Please write the NAME of the most frequently used toothpaste?**	………………………………………
20	**How long do you brush your teeth usually**?	0,5min 1min 2min 3min >
21	**How many Times do you brush your teeth?**	0 1x 2x 3x 4x
22	**What type of toothbrush do you use usually?**	□ manual □ electric □ sonic<br>□ both (manual and other)
23	**How often do you replace for new toothbrush?**	each 2months each 3ms<br>each 6ms 1 a year
24	**During the pandemic, have you noticed a change in your oral hygiene?**	□ NO changes<br>□ YES, I cared for oral hygiene more than before<br>□ YES, I cared for oral hygiene worse than before
25	Do you use dental floss? How many times a day?	□ NO, I do not use<br>□ 1x day<br>□ 2x day<br>□ after each meal
26	**Do you use oral mouthwash? Please indicate the NAME of the most frequently used mouthwash?**	□ NO, I do not use<br>□ YES, I use :………………..
27	**Have you changed the frequency of using mouth rinse during the pandemic?**	□ NO changes<br>□ YES, I rinse more than before<br>□ YES, I rinse less frequent than before
28	**Do you have to use dentures? Which one** (please indicate)	□ YES □ NO<br>□ crowns, bridges<br>□ removable dentures: e.g. acrylic, metallic alloys<br>□ implants
29	**How many years do you use this prosthesis?**	□ NO have □ new □ older how many years?......
30	**Does the prosthesis cause any discomfort (please read and choose examples)?**	□ N/A □ NO □ tightness in the mouth<br>□ pain □ difficulty in eating<br>□ difficulty in speaking □ lack of stabilization
31	**For this reason, in 2020/2021 were you forced to correct the prosthesis yourself?**	□ YES □ NO
32	**Age (years)**	
33	**Height (cm)**	
34	**Body mass (kg)**	
35	**Graduated Education**	□ Primary □ Secondary<br>□ Vocational □ Higher
36	**Sex**	□ MAN □ WOMAN
